# Sentinel Lymph Node Biopsy in Pure DCIS: Is It Necessary?

**DOI:** 10.5402/2012/394095

**Published:** 2012-05-14

**Authors:** D. E. Boler, N. Cabioglu, U. Ince, G. Esen, C. Uras

**Affiliations:** ^1^Department of Surgery, Faculty of Medicine, Acıbadem University, Maltepe, 34848 Istanbul, Turkey; ^2^Department of Pathology, Faculty of Medicine, Acıbadem University, 34848 Istanbul, Turkey; ^3^Department of Radiology, Acıbadem Maslak Hospital, 34457 Istanbul, Turkey; ^4^Department of Surgery, Acıbadem Maslak Hospital, 34457 Istanbul, Turkey

## Abstract

*Introduction.* Sentinel lymph node biopsy (SLNB) in patients with pure ductal carcinoma in situ (DCIS) has been a matter of debate due to very low rate of axillary metastases. We therefore aimed to identify factors in a single institutional series to select patients who may benefit from SLNB. *Material and Methods.* Patients, diagnosed with pure DCIS (*n* = 63) between July 2000 and March 2011, were reviewed. All the sentinel lymph nodes were examined by serial sectioning (50 *μ*m) of the entire lymph node and H&E staining, and by cytokeratin immunostaining in suspicious cases. *Results.* Median age was 51 (range, 30–79). Of 63 patients, 40 cases (63.5%) with pure DCIS underwent SLN, and 2 of them had a positive SLN (5%). In both 2 cases with SLN metastases, only one sentinel lymph node was involved with tumor cells. Patients who underwent SLNB were more likely to have a tumor size >30 mm or DCIS with intermediate and high nuclear grade or a mastectomy in univariate and multivariate analyses. *Conclusion.* In our series, we found a slightly higher rate of SLNB positivity in patients with pure DCIS than the large series reported elsewhere. This may either be due to the meticulous examination of SLNs by serial sectioning technique or due to our patient selection criteria or both.

## 1. Introduction

 Ductal carcinoma in situ (DCIS) of the breast is a preinvasive lesion with theoretically no potential for lymph node or distant metastases by definition. So far the management of axilla in DCIS has dramatically changed. Axillary dissection rates have been decreased since 1990s [1] because it has been shown that omission of axillary dissection in patients with pure in situ disease had no adverse effect on survival or recurrence [1, 2].

In the era of sentinel lymph node biopsy (SLNB) which has been well established in early invasive breast cancer with low axillary recurrence, the center of argument in DCIS has become “to do or not to do SLNB in patients with DCIS?” Today, some authors still believe that there is a subset of patients at high risk for microinvasive disease and subsequent axillary metastasis who may benefit from the SLN biopsy (SLNB) [5, 10–13]. However, there have been reports which discourage SLNB in patients with pure DCIS due to very low rate of axillary metastasis [3, 4, 14–17].

 The flaw is that definitive diagnosis of DCIS cannot be made unless the final histopathological examination is done. However, the surgeon mostly has to decide on whether SLNB is to be performed based on a preoperative stereotactic core-needle biopsy or vacuum aspiration biopsy or tumor characteristics or surgery type. Many surgeons advocate SLNB for patients with DCIS in whom mastectomy was planned to avoid an unnecessary axillary clearance in case an invasive focus has been identified in the final pathology. Presence of high grade DCIS or presence of comedonecrosis, and diagnosis of invasive component by core-needle biopsy, and mammographic DCIS size of at least 4 cm were identified as independent risk factors for invasion [12, 18]. However, no risk factors could be found that were predictive for sentinel lymph node metastasis [18]. Furthermore, presence of comedonecrosis and larger mammographic DCIS size were independent predictors of patients' undergoing SLNB with a presurgical diagnosis of DCIS in core biopsy [12].

 In the present study, we reviewed the patients with a definitive diagnosis of pure DCIS in a single institution series to identify factors to select patients who may benefit from SLNB, and to define our surgical management in these patients.

## 2. Materials and Methods

 Of 699 patients operated for breast cancer at our institution between July 2000 and March 2011, 68 patients (9.7%) were diagnosed with pure DCIS or DCIS associated with microinvasion. Patients with DCIS associated with microinvasion (*n* = 5) were excluded from the study, and 63 patients with pure DCIS were reviewed. The data regarding patient and tumor characteristics were collected form medical records: age at diagnosis, menopausal status, tumor size, nuclear grade, presence of comedo necrosis, type of biopsy (core biopsy, vacuum aspirated biopsy, excisional biopsy by wire-needle localisation or radioguided occult lesion localisation (ROLL)), type of breast surgery (mastectomy versus breast conservation), margin status, estrogen receptor (ER) staining, progesterone receptor (PR) staining, HER2/neu staining, presence of SLNB, and pathological examination of sentinel lymph nodes. The excisional biopsy technique to remove the nonpalpable lesion by ROLL has been described before in detail [19]. The decision on whether a SLNB was to be performed was made for each individual patient separately.

 Lymphatic mapping was done with a combined technique of lymphoscintigraphy after radiocolloid injection at the day of surgery and subareolar methylene blue injection followed by breast massage. All the lymph nodes colored with blue dye and showed radioactivity with gamma probe were removed and were sent for intraoperative evaluation by the pathologist. Briefly, the sentinel lymph node(s) was bisected fresh along its long axis through the hilus or the entering point of afferent lymphatic if it was colored with blue. The node was sliced in 2 mm thickness. Scrape preparations especially from the hilus and from 2 to 4 faces pairs were made and were stained with hematoxylin and eosin (H&E). In the presence of suspicious cells, frozen section from the related slice of the lymph node was made. Finally, the lymph nodes were embedded in paraffin. The entire sentinel lymph nodes were serially sectioned with 50 *μ*m intervals, and two sequential slices with 3 *μ*m thickness were prepared. One of these sequential sections was stained with H&E, and the other one was spared for immunohistochemistry in cases with suspicious atypical cells by H&E to be stained by using a pan-cytokeratin antibody (Novocastra, RTU-PAN-CK, Newcastle, UK). SLN metastases were classified according to the 7th edition of the American Joint Committee on Cancer (AJCC) staging system [20] as follows: isolated tumor cells (ITC) were defined as isolated tumor cells or clusters ≤0.2 mm in maximum diameter; micrometastasis were defined as metastases >0.2 mm but <2 mm; macrometastasis as >2 mm. 

 If the breast lesion consisted of microcalcifications with or without a mass, the entire specimen was sampled after inking and embedded in paraffin after orientation. In cases with a palpable mass, complete sampling of the mass and surgical margins were performed and samples from the surrounding tissue were evaluated for margin evaluation after the specimen was inked. In cases of mastectomy, in addition to complete sampling of microcalcifications or mass, samples of the surrounding tissue and nipple areola complex were also examined. The distance of the tumor to the specimen's inked edge was reported for every marking margin and the margin width was considered as the narrowest distance between the tumor and any inked margin. Microinvasion was defined according to the 7th edition of AJCC staging system [20], which was considered as a microscopic focus of invasion of cancer cells extending beyond the basement membrane into the adjacent tissue, with no focus greater than 0.1 cm in dimension. 

 The histopathologic diagnosis and classification of DCIS were done according to criteria as defined by Rosen and Oberman [21]. Grading of DCIS was categorized as “well-, intermediately, and poorly” differentiated DCIS according to the classification of Holland et al. [22]. Estrogen and progesterone receptor status was evaluated along with HER2/neu protein overexpression as well. Immunostains for ER and PR were performed by using ER (Novocastra (6F11), Newcastle, UK) and PR (Novocastra (PGR-312), Newcastle, UK) antibodies on full sections, and cases with 10% or more positive staining were considered as positive. HER2 positivity was determined based on immunohistochemistry staining by using HER2/neu antibody (Ventana (HER2/neu 4B5), Tucson, Arizona, USA).

The statistical analyses were performed by using the Statistical Package for the Social Sciences (SPSS) program, version 15.0 (SPSS Inc., Chicago, IL, USA). Predictive factors of patients undergoing SLNB were investigated by univariate analyses using Fisher's exact test. The statistically significant variables were further analyzed by logistic regression analyses to identify the independent factors. A *P* value equal or less than 0.05 was considered significant.

## 3. Results

 Sixty-three patients with pure DCIS were reviewed in this study. Median age was 51 (range, 30–79). Thirty-six patients (57.1%) presented with pleomorphic microcalcifications in mammogram without clinical symptom whereas 6 patients (9.5%) presented with pathological nipple discharge and 18 (28.6%) had a palpable mass in physical exam. Two patients (3.2%) had a mass in ultrasound or MRI, whereas one patient had an asymmetric density in the affected breast mammogram. Preoperative diagnosis was made by core biopsy in 12 patients (19%), and by vacuum aspirated biopsy in 4 patients (6.3%), respectively. However, the majority of patients (*n* = 47, 74.6%) underwent excisional biopsy by wire-needle localisation (*n* = 42), or ROLL (*n* = 5) for pathologic diagnosis. Intraoperative frozen section was utilized in 35 cases (55.6%).

 Mastectomy was performed in 32 patients (50.8%) due to multifocal or multicentric disease, whereas 31 patients (49.2%) underwent breast conserving surgery. Forty patients (63.5%) with pure DCIS underwent SLNB, and a median number of 2 SLNs (range, 1–8) were harvested during the procedure. Two patients were found to have a positive SLNB (5%). Of patients with SLN positivity, one patient (2.5%) was demonstrated to have isolated tumor cells (ITCs), whereas one patient had macrometastasis (2.5%). Axillary lymph node dissection was performed in one patient with macrometastasis. In all 2 cases with SLN metastases, only one sentinel lymph node was involved with tumor cells, whereas all the other sentinel and nonsentinel lymph nodes were found to be reactive. The patient with macrometastatis received chemotherapy and adjuvant hormonotherapy for 5 years. The other patient with ITC underwent breast irradiation and received tamoxifen for 5 years. Both of the patients with SLN positivity had high grade tumors, either a palpable mass or a lesion more than 3 cm.

 Patients,who underwent SLNB, were more likely to have a palpable mass, or a tumor size >30 mm or DCIS with intermediate or high nuclear grade or comedonecrosis or mastectomy due to extensive disease (Table 1). Tumor size, nuclear grade, mastectomy, presence of palpable mass were analyzed in logistic regression model. Performing mastectomy, tumor size >30 mm, and presence of intermediate or high nuclear grade were significant independent predictive factors to do a sentinel lymph node biopsy in logistic regression analysis (Table 2). Other factors including age >50, estrogen or progesteron receptor status, or HER-2/neu positivity did not significantly influence the surgeon's decision to perform SLNB.

## 4. Discussion

Although ductal carcinoma in situ (DCIS) is a lesion which has a theoretical risk of 0% for metastases, axillary metastases have been found in 1-2% of the patients treated with axillary dissection [23]. Furthermore, SLN positivity in DCIS is higher than reported axillary positivity ranging between 1% and 13% in published reports as shown in Table 3 [3–5, 9, 24–27]. In a metaanalysis of 3166 patients, the incidence of SLN metastases was 7.4% in patients with a preoperative diagnosis of DCIS compared with 3.7% in patients with a definitive postoperative diagnosis of DCIS alone [13]. Klauber-De More et al. [9] reported 12% SLN positivity in DCIS but when patients with microinvasive focus and patients with stromal and vascular invasion were excluded, the incidence decreased to 6.5%. Pendas et al. [26] reported 4.6% positivity with 4 positive SLNs (by H&E and IHC) in 86 patients with pure DCIS. In a series of 854 patients with pure DCIS, the overall risk of SLN metastases was found to be 1.9% by Intra et al. [3]. This ratio dropped to 1.4% when presence of ITC in SLNs was considered negative according to the last TNM classification [3, 28]. In concordance with some studies [26], we found the SLN positivity as 5% in pure DCIS which is a slightly higher rate of SLN positivity in published large series [3, 13].

The variation in SLN positivity may be attributed to evolution of sentinel node biopsy techniques, different preoperative diagnostic methods, variations in pathological examination including extent of tissue sampling and evaluation of the SLNs with H&E or IHC or both, and small patient numbers in some series [13, 29]. Some reports doubled their node positivity frequencies by using IHC to detect SLN involvement [5, 8, 30]. In a study by Lata et al. [31], in 13% of the patients, SLNs were shown to be involved by tumor cells by IHC methods but no significant association with local, regional or distant recurrence was shown. Wilkie et al. [6] reported metastatic lymph nodes in 5% of 559 patients with a final diagnosis of DCIS after surgical resection and 70% of them were detected only by IHC. In our series, SLNs were meticulously examined with H&E staining after serially sectioned with 50 *μ*m intervals, but IHC was only performed in the presence of suspicious cells.

Most of the SLN metastases of DCIS consist of micrometastases and ITC, and the SLN is the only affected node usually even in the presence of macrometastasis [9, 25, 31]. Similarly, in our series, only one SLN was involved in both cases whereas all the other sentinel and nonsentinel nodes were reactive. This has been mostly attributed to an unrecognized invasive focus in the breast or metastases subsequent to an invasive local recurrence or due to the different examination methods such as H&E or immunohistochemistry [23, 31].

The presence of ITC or micrometastases in the SLN in patients with DCIS is an intriguing issue with unclear clinical implications. Broekhuizen et al. [30] reported an increase in incidence of lymph node metastases after revision with IHC from 1.4% to 10.6% in pure DCIS patients. They mentioned that the cells might have represented a false positive finding associated with microembolism of breast epithelial/tumor tissue that had been dislodged to the lymphatic system by a sampling procedure, but they could not find any evidence of mechanical displacement. However, Bleiweiss et al. [32] demonstrated that CK positive cells in the positive SLNs had different histologic and immunohistochemical characteristics from the primary intraductal carcinoma involving an intraductal papilloma. They suggested that SLNB should not have been a routine procedure until the patient has a histologically proven invasive tumor.

Because of low incidence of the SLN involvement, the routine use of SLNB in pure DCIS is discouraged [3, 14, 16]. However, diagnosis of pure DCIS can only be made after final pathological examination, and in case of an invasive focus in paraffin sections, reoperation for SLNB is needed. It has been reported that an underestimation of an invasive focus is present in 10–42% of patients when preoperative diagnosis is made by core needle biopsy or vacuum aspiration biopsy as in clinical practice [7, 33–38]. When literature that attempted to define a subgroup of patients in whom a second operation for SLNB could be avoided, most of the authors have reported that a palpable mass, mammographic mass, a high grade lesion, and a large size were associated with a significant risk of invasive disease in the final resection specimen despite some inconsistencies between the studies [12, 13, 39–41]. Yen et al. [12] mentioned about 4 independent predictors of invasive cancer on final pathology which were age <55, diagnosis by core needle biopsy, mammographic DCIS size >4 cm, and high grade DCIS. Furthermore, presence of comedonecrosis and larger mammographic DCIS size were found as independent predictors of patients' undergoing SLNB in multivariate analysis. Sakr et al. [7] suggested that DCIS with microinvasion or diffuse DCIS requiring mastectomy (including DCIS more than 30 mm) was the main risk factor for SLN metastasis in a 110-patient series. However, Intra et al. [3] reported that the risk of SLN metastases did not seem to be correlated with the comedocarcinoma subtype, presence of necrosis, tumor grade, hormone receptor status, Ki67, HER2/neu status, multifocality, or type of surgery. The most common architectural patterns were solid and cribriform patterns, but not comedo among those patients with pure DCIS and positive SLNs. The authors found age younger than 50 and mass as clinical presentation to be important factors predicting the likelihood of the SLN metastasis. On the other hand, there have also been some other reports that did not identify any significant predictive risk factors [5, 18, 42, 43].

Our surgical approach to perform SLNB in the same session with definitive operation is in concordance with the published literature [3, 5, 12, 44]. SLNB was not used as a standard procedure in treatment of all DCIS patients. Factors significantly affecting our approach were performing mastectomy, the size of the tumor (>30 mm), and presence of intermediate and high nuclear grade. Patients with large comedo DCIS, large solid tumors, diffuse or multicentric microcalcifications, recurrent lesions, and high grade DCIS were also scheduled for SLNB. In all cases, meticulous examination of the tumor specimen was done to exclude microinvasive foci and to decrease the prevalence of unexpected SLN metastases [10].

## 5. Conclusion

 In our series, we found a relatively higher SLNB positivity in patients with pure DCIS than the large series reported elsewhere. This may either be due to the meticulous examination of SLNs by serial sectioning technique or due to our patient selection criteria or both. Although the importance of presence of ITC in SLNs has not been clarified yet, it may be reasonable to perform SLN in selected patients with pure DCIS. SLNB should be considered in cases of DCIS where there is a strong doubt of invasion at the definitive pathology as in patients with large tumors or diffuse pluricentric microcalcifications undergoing mastectomy or high grade DCIS with comedonecrosis or solid cribriform pattern.

## Figures and Tables

**Table 1:** Univariate analyses for predicting factors associated with the presence of sentinel lymph node biopsy (SLNB) in patients with pure ductal carcinoma in situ (DCIS).

Factors	Use of SLNB (+) (%) (*n* = 40)	*P* value
Age:		0.611
<50 (*n* = 30) versus ≥50 (*n* = 33)	18 (60%) versus 22 (66.7%)	
Palpable mass*:		0.017
(−) versus (+)	23 (44.2%) versus 16 (88.9%)	
Tumor size:		0.006
≤30 mm (*n* = 40) versus >30 mm (*n* = 23)	20 (50%) versus 20 (87%)	
Comedo necrosis*:		0.026
present (*n* = 31) versus absent (*n* = 26)	24 (77.4%) versus 12 (46%)	
Nuclear grade*:		0.012
low (*n* = 15) versus intermediate and high (*n* = 34)	5 (33.3%) versus 25 (73.5%)	
Type of surgery:		0.001
breast conservation (*n* = 31) versus mastectomy (*n* = 32)	13 (41.9%) versus 27 (84.4%)	
Multifocality/multicentricity:		0.182
(−) (*n* = 28) versus (+) (*n* = 34)	21 (75%) versus 19 (55.9%)	
Estrogen receptor status*:		0.622
(+) (*n* = 21) versus (−) (*n* = 7)	17 (81%) versus 5 (71.4%)	
Progesteron receptor status*:		0.634
(+) (*n* = 18) versus (−) (*n* = 10)	15 (83.3%) versus 7 (70%)	
HER2/neu*:		0.364
(IHC 3+) or FISH (+) (*n* = 16) versus other (*n* = 9)	11 (68.8%) versus 8 (88.9%)	

*Unknown data were excluded from the analysis.

**Table 2:** Multivariate analyses for predictive factors associated with the presence of SLNB in patients with pure DCIS.

Factors	Odds ratio (95% CI)	*P* value
Nuclear grade intermediate and high (versus low)	8.1 (1.4–48.2)	0.021
Tumor size >30 mm (versus ≤30 mm)	5.5 (0.9–31.6)	0.059
Mastectomy (versus breast conservation)	14 (2.3–84.3)	0.004
Palpable mass (versus nonpalpable)	3.5 (0.6–21.6)	0.186

**Table 3:** Sentinel lymph node positivity in patients diagnosed with pure ductal carcinoma in situ (DCIS) determined by hematoxylin and eosin (H&E) staining or immunohistochemistry (IHC).

Institution	No. of patients (*n*)	Sentinel lymph node positivity in pure DCIS: by H&E or (IHC)
European Institute of Oncology, Milan [3]	854	1.9%
University of Padova, Italy [4]	102	1%
Lee Moffitt Cancer Center, FL, USA [5]	195	13%: 6.5% by H&E; 6.5% by IHC
Lee Moffitt Cancer Center, FL, USA [6]	559	5%: 1.5% by H&E, 3.5% by IHC
University of Paris, France [7]	110	6%
Sibley Memorial Hospital, Washington DC, USA [8]	110	7.2%: 3.6% by H&E; 3.6% by IHC
Memorial Sloan Kettering Cancer Center [9]	76	12%
Acibadem University, Faculty of Medicine, Istanbul (present study)	40	5%: 2.5% by H&E, 2.5% by IHC
